# Are changes in physical activity during COVID-19 associated with mental health among Danish university students?

**DOI:** 10.3389/fpubh.2023.1126240

**Published:** 2023-04-17

**Authors:** Christina Bjørk Petersen, Christina Krüger, Julie Dalgaard Guldager, Maria Holst Algren, Signe Smith Jervelund, Gabriele Berg-Beckhoff

**Affiliations:** ^1^National Institute of Public Health, University of Southern Denmark, Copenhagen, Denmark; ^2^Unit for Health Promotion Research, Department of Public Health, University of Southern Denmark, Esbjerg, Denmark; ^3^Department of Physiotherapy, University College South Denmark, Esbjerg, Denmark; ^4^Section for Health Services Research, Department of Public Health, University of Copenhagen, Copenhagen, Denmark

**Keywords:** mental health, physical activity, cross sectional, COVID-19, public health, students, youth, Denmark

## Abstract

**Aims:**

The benefits associated with being physical active on mental health is well-established, but little is known on how rapid changes in physical activity are associated with mental health. This study investigated the association between changes in physical activity and mental health among Danish university students during the first COVID-19 lockdown.

**Methods:**

Online survey data were collected among 2,280 university students at the University of Southern Denmark and University of Copenhagen in May–June 2020 as part the “COVID-19 International Student Well-being Study.” Multiple linear regressions were used to analyze associations between changes in physical activity and mental health (depression and stress scores) adjusted for potential socio-economic confounders.

**Results:**

During the first COVID-19 lockdown, 40% decreased their moderate and 44% their vigorous physical activity, while 16% increased their moderate and 13% their vigorous physical activity. Overall, students with a stable physical activity level had the lowest mean depressive and stress scores. Adjusted analyses showed that a decrease in vigorous and moderate physical activity level was significantly associated with a higher depression score (mean difference (vigorous): 1.36, *p* < 0.001 and mean difference (moderate): 1.55, *p* < 0.001). A decrease in vigorous physical activity and an increase in moderate physical activity was associated with a 1-point increase in the PSS-4 stress score (*p* < 0.001).

**Conclusion:**

A substantial proportion of students changed their physical activity level during lockdown. Our findings emphasize the importance of staying physically active during COVID-19 lockdown. This knowledge might be important for relevant health authorities to bridle post-pandemic mental health challenges.

## Introduction

Poor mental health and especially the increase in depressive and anxiety symptoms among adolescents and young people is a critical public health concern since students already before the COVID-19 pandemic experienced higher rates of depressive symptoms than the general population (1). The novel coronavirus (COVID-19) has rapidly altered many facets of life globally. In the spring 2020 during the first wave of COVID-19, governments in Denmark as well as other countries introduced several restrictions to reduce the COVID-19 transmission. In Denmark, the country quickly began to shut down on 12 March 2020 and general restrictions included guidelines for social isolation in case of symptoms, physical distance, hand hygiene, the use of protective equipment in public spaces, and assembly bans (2). Daycare, educational institutions, shops and restaurants/cafes, sport activities and fitness facilities were closed. Thus, universities were closed and converted to digital teaching, and non-critical public employees were required to work from home. Reopening of daycare and younger school classes began on 15 April 2020 (2). From the middle of May all other students in compulsory and upper secondary school returned to school, where most universities continued online teaching until 1 August 2020 (for more detailed information, see (3). In several western countries changes in the physical activity pattern was seen, with a large proportion who decreased and a smaller proportion who increased their physical activity level (4–8). In a stressful situation, such as the COVID-19-pandemic, physical activity may play an important role for maintaining mental well-being.

The association between physical activity and mental health is well established (9). Physically active individuals generally experience less stress, depression, and anxiety (9). Further, studies have shown that physical activity can reduce symptoms of depression (10) and improve mood and well-being (11).

During COVID-19, depressive symptoms and anxiety symptoms have been found to be increased in various age groups in several countries (12). In the general Danish population, mixed results of the impact on mental health were found showing both increased poor mental health (13) and stable mental health reports (14). Among Danish university students, 39% experienced academic distress during lockdown (15). In a number of other countries, there has also been seen a negative impact on mental health among students in terms of stress (16), anxiety (16, 17), depression (16, 18) and distress (17, 18).

Cross-sectional studies have shown a higher degree of depressive symptoms and mood disorders among individuals who decreased their physical activity during the COVID-19 pandemic (4, 8, 19–21). A review from 2020 indicated that a reduction in physical activity levels may have potential long-lasting negative psychological effects (12). However, little is known of the impact of rapid change in physical activity as seen during the lockdown and its relation to mental health among university students. Therefore, the aim of this study was to investigate associations between changes in physical activity level and mental health among Danish university students during the first COVID-19 lockdown. We hypothesized that the COVID-19 lockdown would negatively impact physical activity behavior among university students and that individuals who were physically active during COVID-19 restrictions would report better mental health than those who were not.

## Materials and methods

### Study design

Data was derived from the Danish part of the cross-sectional “COVID-19 International Student Well-being Study” examining the impact of the COVID-19 pandemic on student well-being during the spring of 2020 in 26 countries. The study protocol can be found elsewhere (22). The questionnaire was translated from English into Danish. The translation of the questionnaire from English into Danish was conducted independently by three research staff who thoroughly discussed differences in translation until agreement. A back translation of the survey was not possible due to the time pressure to conduct the survey before the reopening of activities in society was initiated (22). The online survey was distributed predominantly through e-mail to all health and medical science students at the University of Southern Denmark (USD) and the University of Copenhagen (UCPH) as well as to studies of Computer Science, Biology, Economics, Theology, Ethnology, Archaeology, Greek, Latin and History at the UCPH from 11 May to 5 June 2020. The questionnaire was also posted on selected groups in Facebook that included two major dormitories in Copenhagen (Grønjordskollegiet and Egmont), students in political science and psychology as well as one of the author’s own profile (SSJ). The Danish part of the data collection stopped on June 8, 2020.

### Recruitment and study population

A total of 2,945 Danish students participated in the study. Of these, 958 were from the Faculty of Health and Medical Sciences, USD (response rate: 17.8%), 766 students from the Faculty of Health and Medical Sciences, UCPH (response rate: 10.2%) and 1,221 students from other fields of studies and universities (respondents to e-mails from UCPH and Facebook invitations) (3). Participation in the survey was voluntary and all participants provided their consent to participate prior to completing the questionnaire. The study adhered to Danish standards for ethical conduct of scientific studies and was approved by the Research Ethics Committee of the SDU (Case no. 20/29519) and the Independent Ethics Committee for Social Science and Humanities from the University Antwerp in 2020 (Case no. SHW_20_38).

#### Measures

##### Physical activity

Students were asked to indicate their level of physical activity “before the COVID-19 outbreak” and “during the COVID-19 outbreak” in the same questionnaire. The latter was assessed by asking about the activity level during the past week. The student’s physical activity level was measured by moderate and vigorous intensity, separately. On an absolute scale, moderate-to-vigorous physical activity refers to the physical activity that is performed at >3 METs (metabolic equivalent of task) (ie, >3 times the intensity of rest) (23). However, as the MET term is often not used in public health surveillance, moderate-intensity activities was referred to activities that noticeably accelerates the heart rate and vigorous-intensity as activities that result in a significant increase in heart rate. Thus, activities that require a large amount of effort and causes rapid breathing. To guide the participant, examples of moderate and vigorous activities are given in the questionnaire. Moderate physical activity: e.g., easy cycling or walks for at least 30 min. Vigorous activity: e.g., heavy lifting, running, aerobics, or fast cycling with for at least 30 min. Moderate physical activity was measured with the question: “On average, how often did you perform moderate physical activity like easy cycling or walks for at least 30 min?” with five response options: (1) (Almost) never, (2) Less than once a week, (3) Once a week, (4) More than once a week, and (5) (Almost) daily. Vigorous activity was measured with the question: “On average, how often did you perform vigorous physical activity like heavy lifting, running, aerobics, or fast cycling with for at least 30 min?” Response options were similar to those for moderate physical activity. The full questionnaire is available through the Zenodo portal (24) The changes in physical activity were defined as an increase, decrease, or no changes in moderate and vigorous physical activity, during the COVID-19-pandemic as compared to before.

##### Mental health—depressive symptoms and stress

Depressive symptoms were assessed using the modified eight-item version of the Center for Epidemiological Studies Depression Scale (CES-D 8) (25). Validation studies have found good validity and reliability of the scale (CES-D 8: *α* = 0.82 in men and 0.84 in women) (26). The scale consists of eight items: (1) Feeling depressed, (2) Feeling that everything was an effort, (3) Restless sleep, (4) Feeling happy, (5) Feeling lonely, (6) Enjoyed life, (7) Feeling sad, and (8) Unable to get going. On a four-point Likert scale, students ranked how often during the past week the above-mentioned feeling occurred: (1) Rarely or none of the time, (2) Some of the time, (3) Most of the time, or (4) Most of or all the time. For the positively stated questions, four and six, the response rating was reversed. The sum of all the student’s responses made up a score ranging from 5 to 20, with higher scores indicating greater presence of depressive symptoms.

Stress was measured by Cohen’s Short Form Perceived Stress Scale (PSS-4). The PSS-4 is a four-item brief version of the original PSS-14 (27), which has shown a good reliability (*α* = 0.82) (26). The students were asked how often during the past month they felt: (1) Unable to control the important things in life, (2) Confident in the ability to handle personal problems, (3) Things were going your way, or (4) Difficulties were piling up so high that you could not overcome them. Students ranked their responses on a 5-point Likert scale with the following format: (1) Never, (2) Almost never, (3) Sometimes, (4) Fairly often, or (5) Very often. The total score was obtained by reversing responses to the positively stated items and then summing across all scale items. The PSS-4 score rage from 0 to16 with higher scores indicated more perceived stress.

Measurements of depression and stress relating to the students’ mental health were measured only during the COVID-19 outbreak.

##### Covariates—factors associated with changes In physical activity and mental health

Potential confounders were selected *a priori* based on evidence related to physical activity (28) and mental health (21), and inspired by a similar study which investigated changes in physical activity and sedentary behavior and their association with mental health in response to COVID-19 (4). Further, the potential confounders were tested statistically by change in estimates. The following sociodemographic characteristics were included as potential confounders in multiple adjusted analysis: gender, age, relationship status (single, in a relationship, it is complicated), study program (Bachelor, Master or PhD), sufficient income. The following covariates were included in descriptive analysis but not in adjusted analysis: living situation, country of birth (Denmark or elsewhere), changes in income during COVID-19 lockdown and smoking behavior.

### Data analysis

Descriptive analysis involved absolute (*n*) and relative (%) frequency distributions of the sample in relation to physical activity level, mental health and sociodemographic factors using univariate analysis. Changes in physical activity level were examined using Pearson’s chi-squared test and kappa analysis were used to assess the extent of agreement between physical activity level before and during COVID-19 pandemic. Multi-linear regression analyses were performed to assess the association between categories of changes in moderate and vigorous physical activity level and depressive symptoms and stress, respectively adjusting for baseline physical activity level in prior to the COVID-19-pandemic and potential confounding factors (gender, age, relationship status, country of birth, study level, and sufficient income). Residual plots and QQ-plots were used to test assumptions of linearity, homogeneity, and check for outliers. Additionally, sensitivity analyses were conducted including students at the University of Southern Denmark only (19%). These are considered as a more homogenous group since they came predominantly from the Faculty of Health. All significant result were based on a value of *p* of 0.05. Analyses were performed using Stata v. 16.

## Results

Table 1 shows the baseline characteristics of the total study population. In total, 2,945 students participated in the study. Participants with missing information on main variables of interest were excluded leaving a total of 2,280 respondents. Hereof, 78% were women and the mean age was 26 years (std dev: 5.8). About one third were single and 87% were born in Denmark. An equal proportion of the study participants were enrolled in a bachelor program (47%) and a master program (46%), while 7% were PhD students. More than half of the respondents were living with others (58%).

**Table 1:** Description of the study population in the Danish part of the “COVID-19 International Student Well-being Study” carried out in May–June 2020 among 2,280 university students at the University of Southern Denmark and University of Copenhagen.

	*N*	%
Overall *N*	2,280	100.0
*Gender*		
Male	480	21.1
Female	1,785	78.3
Other	15	0.7
*Age*		
<21	263	11.5
22–24	857	37.6
25–30	797	35.0
> 30	363	15.9
*Relationship*		
Single	790	34.7
*Born in Denmark*		
Yes	1,980	86.8
*Study level* *		
Bachelor	1,071	47.0
Master	1,044	45.8
PhD	161	7.1
*Income (before)*		
Sufficient before COVID-19	2,085	91.5
*Income (during COVID-19)*		
Less sufficient during COVID-19	371	16.0
More sufficient during COVID-19	147	6.5
*Living situation (before)*		
With parents	130	5.7
Student hall	314	13.8
With others	1,313	57.6
Alone	449	19.7
Other	74	3.3
*Smoking (before)*		
Yes	249	10.9

* *N* = 4 students with other study level were deleted due to anonymity.

Table 2 show changes in the physical activity level. Prior to the COVID-19 lockdown 82% of the participants were moderately and 58% were vigorously physically active more than once a week. During the lockdown, 33% changed their moderate physical activity level and 46% changes their vigorous physical activity level. In total, 16% increased their moderate physical activity level and 13% increased their vigorous physical activity level, while 40% decreased their moderate physical activity level and 44% decreased their vigorous physical activity level. At follow-up, a lower proportion were physically active as 69% were moderately and 38% were vigorously physically active more than once a week.

**Table 2:** Changes in University students’ average of performing moderate and vigorous physical activity more than once a week before COVID-19 lockdown and during COVID-19 (*n* and cell frequencies).

	Moderate physical activity level during COVID-19							
	<Once a week		Once a week		>Once a week		Total	
Moderate physical activity level before COVID-19*	*N*	%	*N*	%	*N*	%	*N*	%
< Once a week	87	3.8	31	1.4	96	4.2	**214**	**9.4**
Once a week	36	1.6	65	2.9	104	4.6	**205**	**9.0**
> Once a week	219	9.6	267	11.7	1,375	60.3	**1,861**	**81.6**
Total	**342**	**15.0**	**363**	**15.9**	**1,575**	**69.1**	**2,280**	**100.0**
	Vigorous physical activity level during COVID-19							
Vigorous physical activity level before COVID-19**								
< Once a week	471	20.7	43	1.9	95	4.2	**609**	**26.7**
Once a week	164	7.2	85	3.7	90	4.0	**339**	**14.9**
> Once a week	413	18.1	246	10.8	673	29.5	**1,332**	**58.3**
Total	**1,048**	**46.0**	**374**	**16.4**	**858**	**37.6**	**2,280**	**100.0**

* Kappa for moderate physical activity level before and during COVID-19: 0.19; *p* < 0.0001; agreement: 67%.

** Kappa for vigorous physical activity level before and during COVID-19: 0.27; *p* < 0.0001; agreement: 54%. Bold values are the total values - if the preferred, the bold can be left out.

A higher proportion of women than men increased moderate physical activity level during the lockdown period (Supplementary data, Table A1). Participants remaining at a stable level are characterized by a higher proportion being in a relationship, having sufficient and stable income and higher study level. Those who either decreased or increased were more likely to be of younger age, single, study at bachelor level, and experience changes in income. Among those increasing their physical activity level, a higher proportion had increased income during COVID-19 compared with other groups.

### Association between changes in physical activity and mental health

A larger proportion of participants with a high depression score was seen among those who changed their moderate physical activity level, both regarding increased and decreased level (Supplementary data, Table A1). The same pattern was observed for the stress symptoms.

Students with a stable moderate or vigorous physical activity level from prior to during the COVID-19 lockdown had the lowest scores of depression and stress symptoms during the COVID-19 lockdown (CES-D 8 mean score: 7.5 and PSS 4 mean score: 6.1 for stable moderate physical activity level; Table 3). Compared to a stable level, adjusted analysis show that a decrease in moderate or vigorous physical activity was significantly associated with a higher scores of depression symptoms during the COVID-19 lockdown (mean difference in CES-D 8 depression score: 1.35, *p* < 0.001 for vigorous and 1.56, *p* < 0.001 for moderate). No difference between stable and increased vigorous physical level was observed in adjusted analysis although unadjusted analysis shows a significant lower score for an increase in vigorous physical activity and a higher score for an increase in moderate physical activity.

**Table 3:** The association between changes in moderate and vigorous physical activity and depression and stress scores with accompanying *value of p* and 95% confidence interval, *n* = 2,280.

					Unadjusted			Adjusted^6^		
Change in physical activity (PA) level		No. of students	Mean score (std. dev.)	95%CI	Diff.^5^	*p*-Value	95%CI	Diff.^5^	*p*-Value	95%CI
		Depression scores (CES-D 8)								
Moderate PA^1^										
	No change (ref.)	1,001	7.5 (4.3)	7.28; 7.80	-	-	-	-	-	-
	Increase	368	8.7 (4.6)	8.24; 9.19	1.18	<0.001	0.65; 1.72	0.39	0.208	−0.22; 1.00
	Decrease	911	9.4 (4.6)	9.06; 9.66	1.82	<0.001	1.42; 2.22	1.56	<0.001	1.17; 1.96
										
Vigorous PA^2^										
	No change (ref.)	964	7.7 (4.4)	7.46; 8.01	-	-	-	-	-	-
	Increase	307	8.5 (4.4)	8.00; 9.00	0.76	0.010	0.19; 1.34	- 0.01	0.994	−0.57; 0.57
	Decrease	1,009	9.1 (4.6)	8.85; 9.42	1.40	<0.001	1.00; 1.80	1.35	<0.001	0.95; 1.76
										
		Stress scores (PSS-4)								
Moderate PA^3^										
	No change (ref.)	1,001	6.1 (3.2)	5.91; 6.30	-	-	-	-	-	-
	Increase	368	7.0 (3.3)	6.69; 7.35	0.91	<0.001	0.52; 1.30	0.45	0.041	0.02; 0.89
	Decrease	911	7.2 (3.3)	6.97; 7.40	1.08	<0.001	0.79; 1.37	0.91	<0.001	0.62; 1.20
										
Vigorous PA^4^										
	No change (ref.)	964	6.2 (3.2)	6.00; 6.40	-	-	-	-	-	-
	Increase	307	6.7 (3.2)	6.37; 7.09	0.52	0.014	0.11; 0.94	0.05	0.796	−0.36; 0.47
	Decrease	1,009	7.1 (3.3)	6.92; 7.34	0.93	<0.001	0.64; 1.21	0.86	<0.001	0.57; 1.16
										

1 *r*^2^ = 0.08 (adjusted model).

2 *r*^2^ = 0.11 (adjusted model).

3 *r*^2^ = 0.08 (adjusted model).

4 *r*^2^ = 0.07 (adjusted model).

5 Difference in depression score due to changes in vigorous and moderate physical activity, respectively.

6 Moderate physical activity: association adjusted for moderate physical activity before COVID-19, age, gender, relationship status, study program, and insufficient income. Vigorous physical activity: association adjusted for vigorous physical activity before COVID-19, age, gender, relationship status, study program, and sufficient income.

A decrease in vigorous and moderate physical activity level was associated with a 1-point increase in the PSS-4 stress score (PSS 4 mean score: 7.1 for decrease in vigorous and 7.2 for decrease in moderate). This association was also significant in the multiple adjusted analysis. In unadjusted models, a higher mean stress score was observed for those who increased physical activity level. After adjustments, this association remained significant for an increase in moderate (*p* = 0.041) but not for vigorous physical activity level (*p* = 0.796). Sensitivity analysis including students at the University of Southern Denmark showed essentially same results, although not significant due to the lower number of respondents (Supplementary data, Table A2).

## Discussion

In this cross-sectional national survey of Danish university students, a third of the participants changed their moderate physical activity level and almost half of the participants changed their vigorous physical activity level during the COVID-19 lockdown. Only a small proportion increased their physical activity level. Overall, findings suggest that students who were physically active prior to the COVID-19 lockdown and reported no change in their physical activity level during the lockdown seemed less affected mentally compared with those who changed their physical activity level. A change in physical activity level was associated with higher scores of stress and depression symptoms. Compared to a stable physical activity level, students reporting a decrease in physical activity level were more likely to have higher scores in depressive and stress symptoms, but a higher stress score was also seen among those who increased their moderate physical activity level.

### Changes in Danish student’s physical activity level during the COVID-19 lockdown in 2020

Our finding of a large proportion of students who changed, and lowered, their physical activity level during lockdown is in line with findings from other studies (4–7, 19, 20). For example, a considerable reduction of physical activity was observed among Italian undergraduate students during the period of lockdown (5) and among Spanish adults a decrease in physical activity was observed especially among young people, students, and very active men (6).

We found an 18% increase in the proportion of students performing moderate physical activity more than once a week during the lockdown. It is possible that the lockdown afforded the opportunity to engage in some physical activities such as walking during breaks or other activities causing a positive change in physical activity behavior for some students. Results from a multi-country study from New Zealand, Australia, and the UK indicated that the lockdown period did allow for some individuals to engage in more daily physical activity and that 74% of these were individuals who did not meet recommended PA guidelines before COVID-19 (20). However, our results indicate that some students were more likely to change physical activity behavior during the lockdown compared to others. In general, those who remained stable were older and more experienced students and for this reason their physical activity behavior be less influenced the lockdown. Also, it may indicate that the stability in their educational program and their relationship enforce stability in physical activity behaviors.

### Association between changes in physical activity with mental health

Experimentally studies have shown a significant impact on depression and mood after just 1 week of physical inactivity among active adults (10, 11), which is aligned with our findings. Similarly, our findings converge with results from other cross-sectional studies showing a higher degree of depressive symptoms and mood disorders among individuals who decrease physical activity during the COVID-19-pandemic (4, 8, 19–21). For example, a study among 3,052 US adults, showed that those who were physically active but decreased physical activity during lockdown had stronger/higher depressive symptoms compared to those who maintained adherence to physical activity guidelines (4).

Possible explanations for the beneficial effect of physical activity on mental health are for example changes in self-perceptions, self-esteem as well as neurobiological and behavioral mechanisms (29). Specific to COVID-19, moderate- and high-intensity physical activity have been suggested to induce not only positive effect on the immune (30), but also on the neurological system (31). The latter may explain why exercise can attenuate depression levels (31) as seen in a study among Vietnamese adults with suspected COVID-19 symptoms (32).

The observed association between increased moderate physical activity level and higher stress score during the lockdown period does not match the overall body of research showing that regular physical activity is associated with better mental health (33). However, the cross-sectional nature of this study introduces a risk of reverse causation. Students who experience depression and stress symptoms during the lockdown may engage more frequently in physical activities as a self-management strategy to counter their conditions. Thus, the cross-sectional associations showing a worsening in symptoms should not be interpreted as an effect of increased physical activity. Supporting evidence from other studies shows that people who were physically active during lockdown were less likely to report depressive and anxiety symptoms (21), and lower levels of mental health (34).

It is possible that isolation/quarantine was associated with lower mental health regardless of changes in physical activity behavior. Closing physical activities and sport clubs hamper not only physical activity levels but also social interactions, which is likely to impact mental health and explain the observed findings. Furthermore, the lockdown increased screen-based work and teaching, which may be associated with lower mental health (19).

### Strengths and limitations

First, given the cross-sectional nature of our work, it is not possible to make causal inference of the associations. Thus, even though the findings overall match the existing body of research showing that regular physical activity is associated with better mental health, it is essential to further examine the observed associations in longitudinal designs. Also, although we adjusted for relevant potential confounders, potential confounders such as absence of mental health level of students before the pandemic or of history of mental conditions as well as academic year were not adjusted for. Second, self-reported measures were used to measure physical activity. Participants were asked retrospectively about their pre-lockdown physical activity and therefore their answers rely on recall of behaviors. Students with impaired mental health may recall their activity levels prior to COVID-19 differently than students with less affected mental health. A third possible bias concerns the sample. Despite having a moderate sample size, the sample was recruited *via* e-mail and Facebook, which may not be representative for the entire population. This is vivid in the sample consisting of mainly female health and medical science students.

Strengths include the timing of the survey, which was at the end of the first Danish lockdown where the reaction in the population is assumed to be most significant. Also, the target group stands out. University students are often overseen in research and during the past decades there is seen a pattern of worsening mental health among European youths which has accelerated during the COVID-19-pandemic (15, 17, 18). While much focus has been on the deleterious effects of lockdown on mental health, our findings point to the importance of investigating the association between changes in physical activity during the COVID-19-pandemic and mental health. In times of social distancing and lockdown, our study supports a strengthen focus on public health efforts and strategies to promote opportunities for regular physical activity to preserve good mental health among university students.

## Conclusion

We found a substantial change in Danish university students’ physical activity habits during the COVID-19 lockdown and that these changes were associated with their mental health. A decrease in moderate and vigorous physical activity level was associated with more depression and stress symptoms. The current findings support the mental health benefits of physical activity. In the wake of COVID-19 or for future pandemics It emphasizes a need for effective public health strategies to promote physical activity to bridle a mental health crisis. Future research should examine changes in physical activity and its impact on mental health in longitudinal designs.

## Data availability statement

Publicly available C19 ISWS datasets were analyzed in this study. The C19 ISWS adheres to the principle of ‘FAIR Data’, by making the data freely available to all C19 ISWS partners within the first year, and making the data freely accessible to all researchers 1 year after closing of data collection. To stimulate the use of the C19 ISWS data and to encourage research collaborations, the project was registered on the World Pandemic Research Network, while the questionnaire and (preliminary) research results are accessible through the Zenodo portal: https://zenodo.org/.

## Ethics statement

Ethical review and approval was not required for the study on human participants in accordance with the local legislation and institutional requirements. The patients/participants provided their written informed consent to participate in this study.

## Author contributions

CP, CK, and GB-B: conceptualization the research question for the current study. JG, SJ, and GB-B: conceptualization and conducting the data collection. CK and GB-B: formal analysis. CP and CK: writing—original draft preparation. JG, MA, SJ, and GB-B: writing—review and editing. All authors contributed to the article and approved the submitted version.

## Conflict of interest

The authors declare that the research was conducted in the absence of any commercial or financial relationships that could be construed as a potential conflict of interest.

## Publisher’s note

All claims expressed in this article are solely those of the authors and do not necessarily represent those of their affiliated organizations, or those of the publisher, the editors and the reviewers. Any product that may be evaluated in this article, or claim that may be made by its manufacturer, is not guaranteed or endorsed by the publisher.
