# Supplementary material for: Are changes in physical activity during COVID-19 associated with mental health among Danish university students?
Source: Front Public Health. 2023 Apr 17;11:1126240. doi: 10.3389/fpubh.2023.1126240 (PMC10149910; doi:10.3389/fpubh.2023.1126240)
Supplement: Supplementary file 1 [file Table_1.DOCX]

Supplementary Material

# Are changes in physical activity during COVID-19 associated with mental health among Danish university students?

Christina Bjørk Petersen^1*^, Christina Krüger^2^, Julie Dalgaard Guldager^2,3^, Maria Holst Algren^1^, Signe Smith Jervelund^4^, Gabriele Berg-Beckhoff^2^.

^1^ National Institute of Public Health, University of Southern Denmark, Copenhagen, Denmark.

^2^ Unit for Health Promotion Research, Department of Public Health, University of Southern Denmark, Esbjerg, Denmark

^3^ Department of Physiotherapy, University College South Denmark, Esbjerg, Denmark

^4^ Section for Health Services Research, Department of Public Health, University of Copenhagen, Denmark

*** Correspondence:**Christina Bjørk Petersen

National Institute of Public Health, University of Southern Denmark, Copenhagen, Denmark.

e-mail: chrb@sdu.dk

**
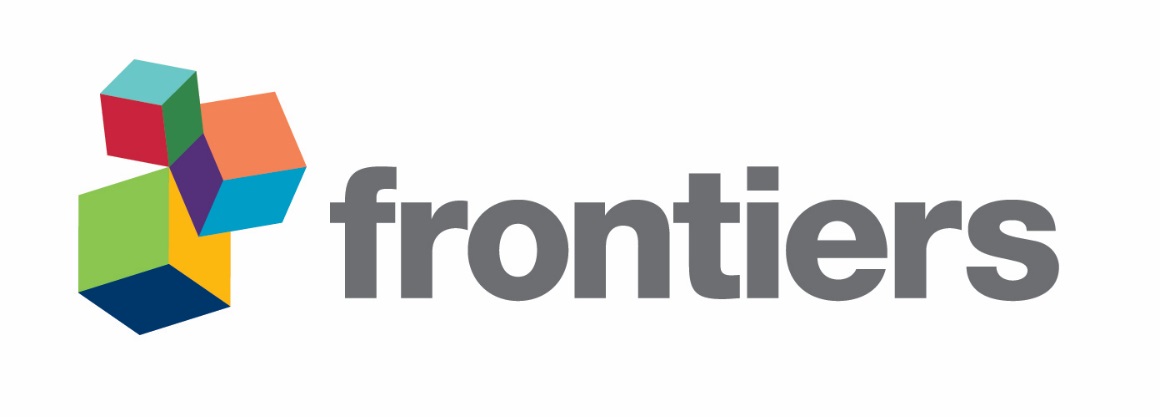
**

**Table A1.** Characteristics (number and percentage) among all 2.280 participants University Students according to changes in physical activity level during the first COVID-19 lockdown in Denmark

|  | *Overall* | | *No change in moderate PA* | | *Increase in moderate PA* | | Decrease in moderate PA | |
| --- | --- | --- | --- | --- | --- | --- | --- | --- |
|  | *N* | % | *N* | % | *N* | % | *N* | % |
| Overall *N* | 2280 | 100.0 | 1,001 | 43.9 | 368 | 16.1 | 911 | 40 |
| Gender  *Male*  *Female*  *Other* | 480  1,785  15 | 21.1  78.3  0.7 | 226 766 9 | 22.6 76.5 0.9 | 58 309 1 | 15.8 84.0 0.3 | 196 710 5 | 21.5 77.9 0.6 |
| Age  *<21*  *22-24*  *25-30*  *> 30* | 263  857  797  363 | 11.5  37.6  35.0  15.9 | 88 346 362 205 | 8.8 34.6 36.2 20.5 | 51 145 122 50 | 13.9 39.4 33.2 13.6 | 124 366 313 108 | 13.6 40.2 34.4 11.9 |
| Relationship  *Single*  *In a relationship*  *It is complicated* | 790  1,420  70 | 34.7  62.3  3.1 | 326  653  22 | 32.6  65.2  2.2 | 135  218  15 | 36.7  59.2  4.1 | 329  549  33 | 36.1  60.3  3.6 |
| Born in Denmark  *Yes* | 1,980 | 86.8 | 880 | 87.9 | 317 | 86.1 | 783 | 86.0 |
| Depression score  *Low*  *Middle*  *High* | 862 745 673 | 37.8 32.7 29.5 | 455 326 220 | 45.4 32.6 22.0 | 128 125 115 | 34.8 34.0 31.2 | 279 294 338 | 30.6 32.3 37.1 |
| Stress score  *Low*  *Middle*  *High* | 860 746 674 | 37.7 32.7 29.6 | 447 310 244 | 44.6 31.0 24.4 | ‘  117 139 112 | 31.8 37.8 30.4 | 296 297 318 | 32.5 32.6 34.9 |
| Study level*  *Bachelor*  *Master*  *PhD* | 1,071  1,044  161 | 47.0  45.8  7.1 | 402 500 96 | 40.2 50.0  9.6 | 187 161 20 | 50.8 43.8 5.4 | 482 383 45 | 52.9 42.0 4.9 |
| Income (before)  *Sufficient* | 2085 | 91.5 | 931 | 93.0 | 322 | 87.5 | 832 | 91.3 |
| Income (during COVID-19)  *Less than before*  *More than before* | 371  147 | 16.0  6.5 | 136 57 | 13.6 5.7 | 67 33 | 18.2 9 | 168 57 | 18.4 6.3 |
| Living situation (before)  *With parents*  *Student hall*  *With others*  *Alone*  *Other* | 130  314  1,313  449  74 | 5.7  13.8  57.6  19.7  3.3 | 53 125 600 188 35 | 5.3 12.5 59.9 18.8 3.5 | 26 54 209 68 11 | 7.1 14.7 56.8 18.5 3.0 | 51 135 504 193 28 | 5.6 14.8 55.3 21.2 3.1 |
| Smoking (before)  *Yes* | 249 | 10.9 | 102 | 10.2 | 29 | 7.9 | 118 | 13.0 |

**Table A2**. The association between changes in moderate and vigorous physical activity and depression and stress scores with accompanying *p*-value and 95% confidence interval, *n*= 2,280.

|  |  | | | | Unadjusted | | | Adjusted^6^ | | |  |
| --- | --- | --- | --- | --- | --- | --- | --- | --- | --- | --- | --- |
| **Change in physical activity (PA) level** | | No. of students | Mean score (std. dev.) | 95%CI | Diff.^3^ | *P*-value | 95%CI | Diff.^5^ | *P*-value | 95%CI |  |
|  |  | **Depression scores (CES-D 8)** | | | | | | | | | |
| **Moderate PA^1^** | |  |  |  |  |  |  |  |  |  |  |
|  | No change (ref.) | 459 | 6.9 (4.1) | 6.52; 7.28 | - | - | - | - | - | - |  |
|  | Increase | 166 | 8.2 (4.7) | 7.33; 9.06 | 1.28 | 0.001 | 0.50; 2.06 | 0.68 | 0.117 | -0.17; 1.53 |  |
|  | Decrease | 367 | 8.7 (4.6) | 8.23; 9.17 | 1.82 | <0.001 | 1.22; 2.42 | 1.29 | <0.001 | 0.69; 1.89 |  |
|  |  |  |  |  |  |  |  |  |  |  |  |
| **Vigorous PA^2^** | |  |  |  |  |  |  |  |  |  |  |
|  | No change (ref.) | 428 | 7.1 (4.2) | 6.70; 7.50 | - | - | - | - | - | - |  |
|  | Increase | 115 | 8.1 (4.7) | 7.23; 8.97 | 0.98 | 0.035 | 0.07; 1.89 | 0.22 | 0.643 | -0.69; 1.13 |  |
|  | Decrease | 449 | 8.4 (4.5) | 7.98; 8.82 | 1.32 | <0.001 | 0.74; 1.91 | 0.95 | 0.002 | 0.34; 1.56 |  |
|  |  |  |  |  |  |  |  |  |  |  |  |
|  |  | **Stress scores (PSS-4)** | | | | | | | | |  |
| **Moderate PA^3^** | |  |  |  |  |  |  |  |  |  |  |
|  | No change (ref.) | 1,001 | 6.1 (3.2) | 5.91; 6.30 | - | - | - | - | - | - |  |
|  | Increase | 368 | 7.0 (3.3) | 6.69; 7.35 | 0.91 | <0.001 | 0.52; 1.30 | 0.45 | 0.041 | 0.02; 0.89 |  |
|  | Decrease | 911 | 7.2 (3.3) | 6.97; 7.40 | 1.08 | <0.001 | 0.79; 1.37 | 0.91 | <0.001 | 0.62; 1.20 |  |
|  |  |  |  |  |  |  |  |  |  |  |  |
| **Vigorous PA^4^** | |  |  |  |  |  |  |  |  |  |  |
|  | No change (ref.) | 428 | 5.7 (3.0) | 5.41; 5.99 | - | - | - | - | - | - |  |
|  | Increase | 115 | 6.1 (3.2) | 5.51; 6.69 | 0.35 | 0.278 | 0.11; 0.94 | 0.05 | 0.796 | -0.36; 0.47 |  |
|  | Decrease | 449 | 6.5 (3.2) | 6.20; 6.80 | 0.82 | 0.000 | 0.64; 1.21 | 0.86 | 0.000 | 0.57; 1.16 |  |
|  |  |  |  |  |  |  |  |  |  |  |  |
| 1. r^2^ = 0.08 (adjusted model)  2. r^2^ = 0.11 (adjusted model)  3. r^2^ = 0.06 (adjusted model)  4. r^2^ = 0.06 (adjusted model)  5. Difference in depression score due to changes in vigorous and moderate physical activity, respectively.  6. Moderate physical activity: association adjusted for moderate physical activity before COVID-19, age, gender, study program, and insufficient income. Vigorous physical activity: association adjusted for vigorous physical activity before COVID-19, age, gender, relationship status, study program, and sufficient income. | | | | | | | | | | | |
